# Anthocyanins: A Comprehensive Review of Their Chemical Properties and Health Effects on Cardiovascular and Neurodegenerative Diseases

**DOI:** 10.3390/molecules25173809

**Published:** 2020-08-21

**Authors:** Roberto Mattioli, Antonio Francioso, Luciana Mosca, Paula Silva

**Affiliations:** 1 Department of Sciences, RomaTre University, v.le G. Marconi 446, 00146 Rome, Italy; roberto.mattioli@uniroma3.it; 2 Department of Biochemical Sciences, Sapienza University, p.le Aldo Moro, 5, 00185 Rome, Italy; antonio.francioso@uniroma1.it; 3 Laboratory of Histology and Embryology, Institute of Biomedical Sciences Abel Salazar (ICBAS), Rua de Jorge Viterbo Ferreira n°228, 4050-313 Porto, Portugal

**Keywords:** anthocyanins, anthocyanidins, bioavailability, antioxidants, colorants, biological activity, biosynthesis

## Abstract

Anthocyanins are a class of water-soluble flavonoids widely present in fruits and vegetables. Dietary sources of anthocyanins include red and purple berries, grapes, apples, plums, cabbage, or foods containing high levels of natural colorants. Cyanidin, delphinidin, malvidin, peonidin, petunidin, and pelargonidin are the six common anthocyanidins. Following consumption, anthocyanin, absorption occurs along the gastrointestinal tract, the distal lower bowel being the place where most of the absorption and metabolism occurs. In the intestine, anthocyanins first undergo extensive microbial catabolism followed by absorption and human phase II metabolism. This produces hybrid microbial–human metabolites which are absorbed and subsequently increase the bioavailability of anthocyanins. Health benefits of anthocyanins have been widely described, especially in the prevention of diseases associated with oxidative stress, such as cardiovascular and neurodegenerative diseases. Furthermore, recent evidence suggests that health-promoting effects attributed to anthocyanins may also be related to modulation of gut microbiota. In this paper we attempt to provide a comprehensive view of the state-of-the-art literature on anthocyanins, summarizing recent findings on their chemistry, biosynthesis, nutritional value and on their effects on human health.

## 1. Introduction

Increased lifespan and a better quality of life have dramatically improved life expectancy of the world population. However, wide availability of hypercaloric foods and the increase in consumption of processed foodstuff, particularly in the western countries, has led to the epidemic of chronic non communicable diseases such as cardiovascular, metabolic, and neurodegenerative diseases. In this perspective, the consumption of fresh foods containing non-nutrient bioactive compounds should be promoted, as they provide health protection at many different levels. Indeed, fresh foods, particularly plant food, contain a plethora of bioactive compounds such as polyphenolic compounds, which are able to modulate different pathways and processes in our body and display antioxidant, anti-inflammatory, anticancer, glucose regulating and neuroprotective activities.

Among polyphenols, an interesting class of compounds is represented by anthocyanins. These compounds are water soluble vacuolar pigments present mostly in fruits and flowers, but also in vegetative organs. They have a strong impact on food sensory properties, as they provide the characteristic red to blue color in fruits and vegetables. In plants, they play a key role in pollination and by absorbing light, protect plants from UV ray-induced damage and from cold stress [1,2,3]. The color of some organs, such as the petals of the flowers, can change during development both through the synthesis of a greater or lesser amount of anthocyanins and through a different acidification of the vacuole [4]. While the variation of anthocyanin concentration alters the color intensity, the different vacuolar acidification changes the hue [5]. The main function of the anthocyanins, contained in flowers or in fruit epidermis, is to attract animals and pollinating insects to easily disseminate the seeds or to facilitate the spread of pollen. However, evidence that the synthesis of anthocyanins is induced during the establishment of adverse conditions suggests their involvement also in both biotic and abiotic stresses.

Anthocyanins are glucosides of the anthocyanidins, flavonoid derivatives produced via the phenylpropanoid pathway. They are present in all tissues of higher plants, including leaves, stems, roots, flowers, and fruits. The six predominant anthocyanidins found in foods are cyanidin, delphinidin, pelargonidin, peonidin, petunidin, and malvidin [6,7].

Anthocyanin pigments have been widely used as natural food colorants. However, the color and stability of these pigments are influenced by pH, light, temperature, and structure. At acidic pH, anthocyanins are red pigments, while they shift to blue in a basic environment. However, at basic pH, anthocyanins are unstable and tend to degrade to dark brown oxidized compounds [6]. Anthocyanin stability also depends on the B-ring in their structure and on the presence of hydroxyl or methoxyl groups. Indeed, the presence of an oxonium ion adjacent to carbon 2 makes the anthocyanins particularly susceptible to nucleophilic attack by compounds like sulfur dioxide, ascorbic acid, hydrogen peroxide or water. The presence of metal ions, temperature, light and oxygen may also affect their stability [6].

Besides the use as food colorants, these compounds are potentially useful as nutraceutical ingredients, as they provide numerous beneficial health effects. Many in vitro, animal and human studies have evaluated the biological and pharmacological potential of these molecules and demonstrated that they possess the capacity to counteract oxidative stress, to act as antimicrobial substances, and to counteract the onset and progression of numerous non-communicable diseases such as neurodegenerative, cardiovascular, metabolic diseases and cancer [6]. They are also well known because they protect visual function along with vitamin A and carotenes [8]. The activities of anthocyanins have been attributed to their free-radical scavenging capacity and to their action on an array of enzymes like cyclooxygenase and mitogen-activated protein kinase, and on inflammatory cytokines signaling. No negative effect of anthocyanin derivatives has been reported, even after ingestion of very high doses, hence their use in the prevention or treatment of numerous diseases is an appealing possibility. Here, we make an attempt to review the most recent literature on the chemistry and biochemistry of these very interesting and potentially helpful pigments.

## 2. Chemistry and Biochemistry of Anthocyanins

### 2.1. Structural Determinants of Anthocyanins

**Figure 1:**
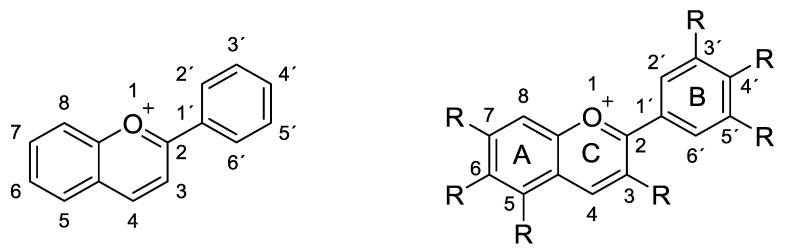
Chemical structure of the flavylium cation on the left and of anthocyanidin backbone on the right with atom numbering and ring label (R = H, OH).

Anthocyanins are the glycosylated forms of anthocyanidins (aglycones). These compounds are formed by a flavylium cation backbone hydroxylated in different positions (generally on carbons C3, C5, C6, C7 and C3’, C4´, C5´) to give rise to different anthocyanidins (Figure 1). Even if these molecules contain an oxonium group in their structure, the flavonoid skeleton maintains its ring nomenclature with the charged oxygen atom on the C ring [1].

**Figure 2:**
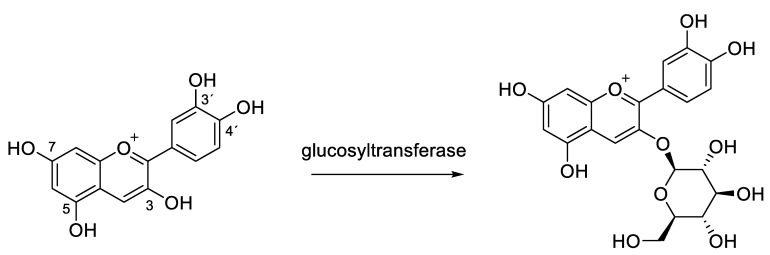
Cyanidin (anthocyanidin, **left**) and its 3-*O*-glycosyl product chrysanthemin (anthocyanin, **right**) derived from the enzymatic activity of glucosyltransferase.

Glycosylation of anthocyanidins to form the respective anthocyanins can occur on different hydroxyl moieties of the molecule with 3-OH as the most abundant glycosylation site in nature to produce 3-*O*-*β*-glucosides (e.g. Chrysanthemin from cyanidin, Figure 2 [7,9].

**Figure 3:**
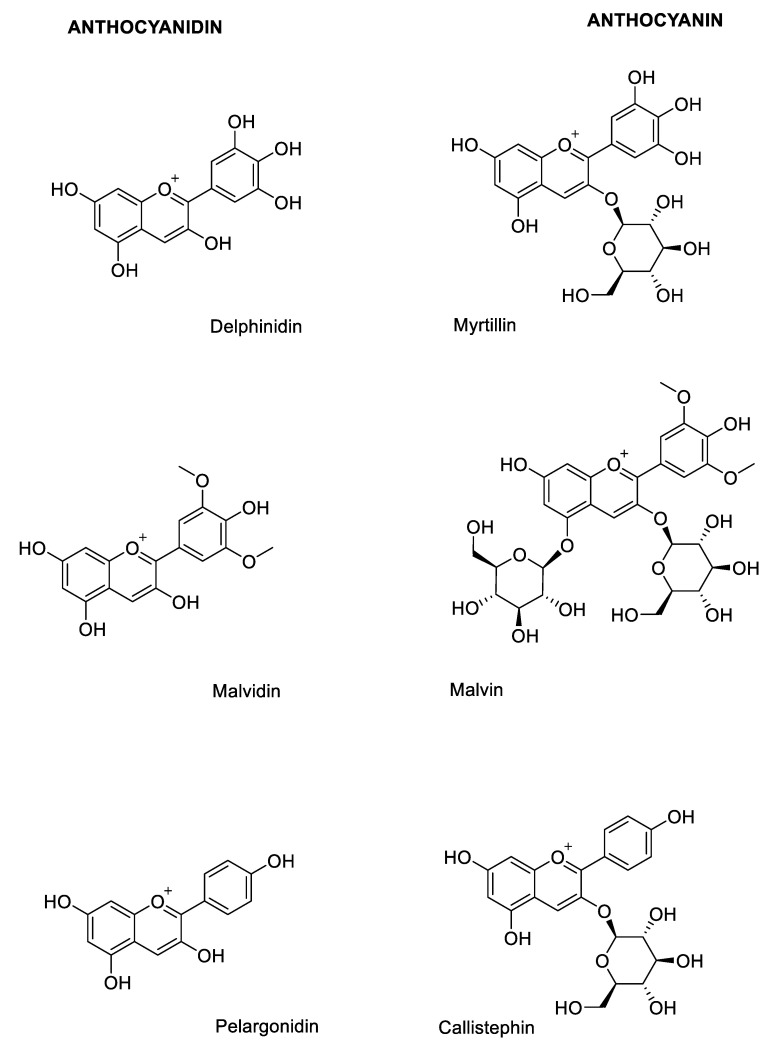
Chemical structure of some naturally occurring anthocyanidins (**left**) and corresponding mono- or di-glycosylated anthocyanins (**right**).

Among most frequently naturally occurring monomeric anthocyanins, there are the glycosides of cyanidin, delphinidin, malvidin and pelargonidin (Figure 3) [10,11].

**Figure 4:**
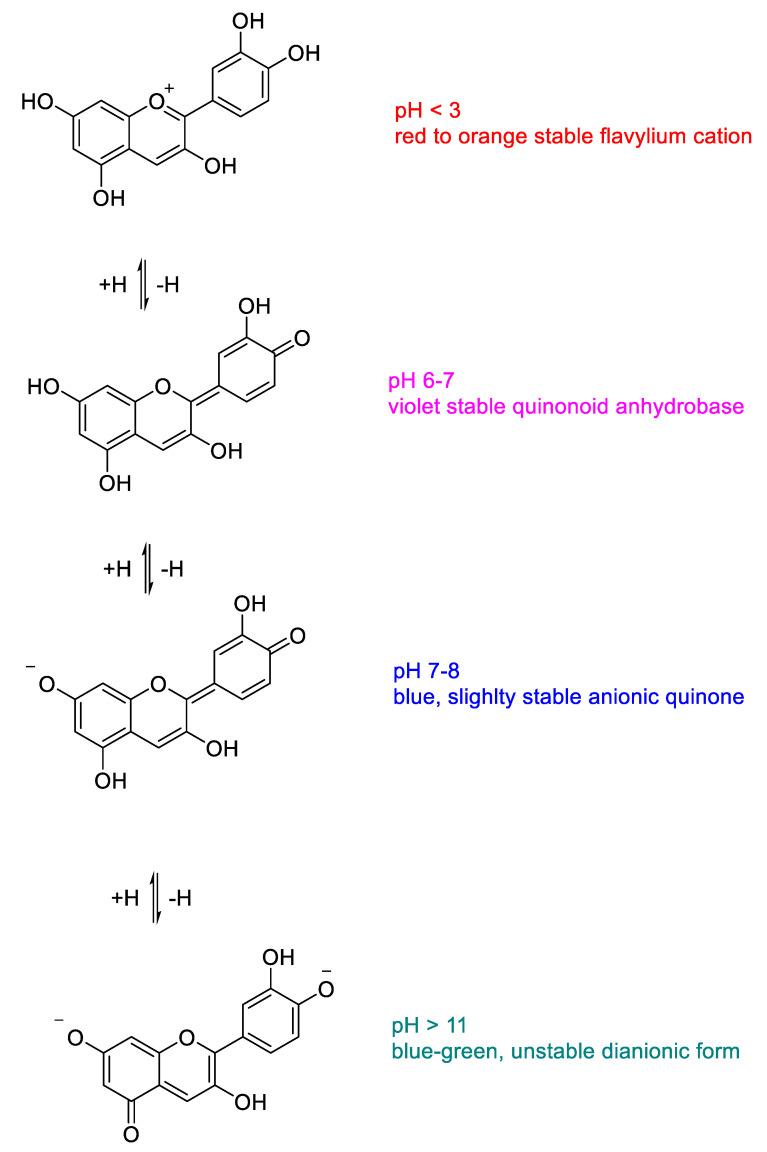
Molecular states and electronic delocalization of cyanidin at different pH values.

These compounds display different colors (red, blue and purple) depending on their accumulation and chlorophyll complementary light absorbance. Their ability to shift the typical green pigment is an important protective mechanism in some plants. Light absorbance, pH-dependent coloration and stability of anthocyanins are strictly related features, all of them involving the electronic conjugation properties around the oxonium moiety that characterize this class of compounds. The colors that some plants (especially flowers and fruits) can assume are in many cases the result of the combined light absorbance of chlorophylls and anthocyanins [1,7,9]. As mentioned above, these color changes can be a defense mechanism of some plants that are able to attenuate their strong attractive green coloration, protecting themselves from possible dangerous herbivorous predators [12,13]. Intrinsic anthocyanidin and anthocyanin colors are related to their UV-visible spectral absorption, electronic conjugation and delocalization properties. These effects are induced by the different ionization states and electronic rearrangements in the molecules that are strongly influenced by the protonic concentrations in the environment [1,9,14]. At low pH values, anthocyanins are present as flavylium cations (oxonium charged oxygen), while at neutral conditions uncharged quinones are formed. At basic conditions, all anthocyanins are slightly stable (a feature that increases proportionally with the pH) and can undergo different degradation pathways with subsequent loss of coloration (Figure 4).

### 2.2. Antioxidant Activity

**Figure 5:**
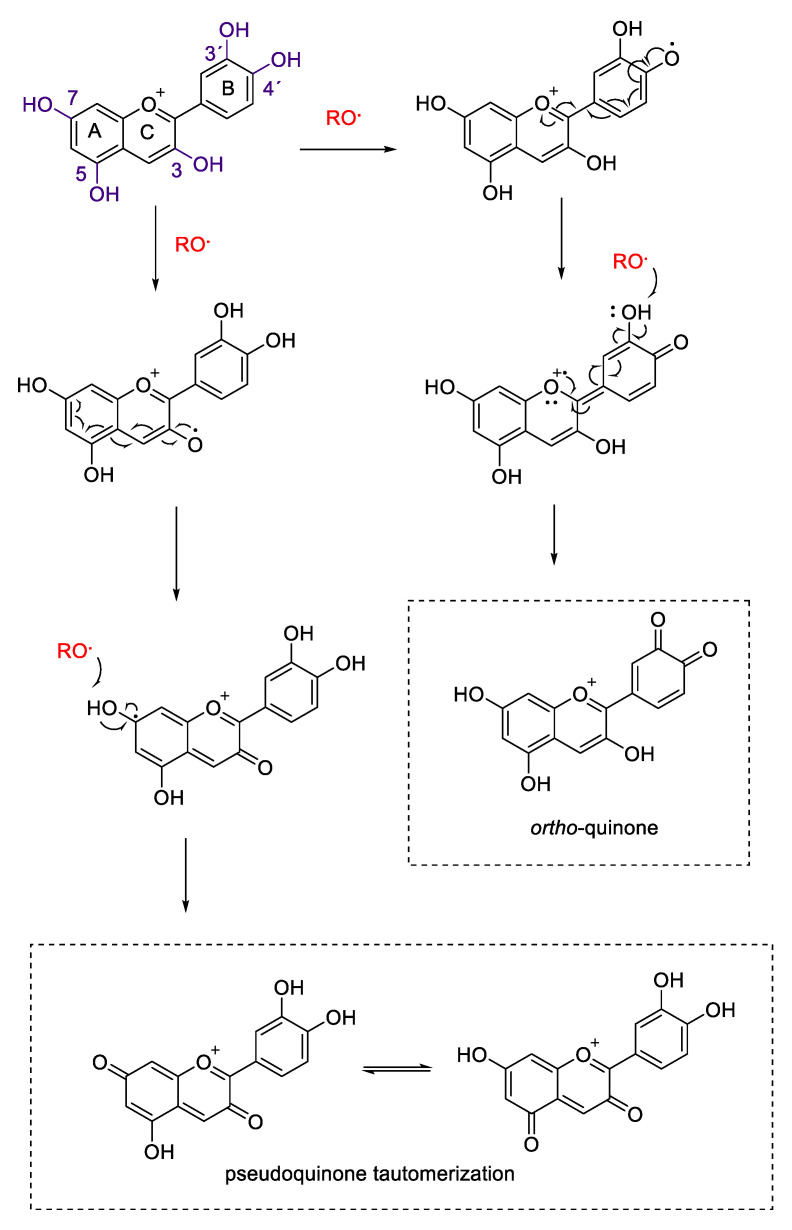
Cyanidin antioxidant scavenging mechanisms against a generic radical oxidant (RO•). Radical attack on position 3 (**left**) and 4´ (**right**) are shown with the respective electron delocalization and resonance structures.

Anthocyanins and anthocyanidins, as other polyphenols and flavonoids, possess the ability to act as free radical scavengers against harmful oxidants such as reactive oxygen and nitrogen species (ROS and RNS) [15]. The flavylium skeleton provides anthocyanins with particular features involving radical electron delocalization on sp2 orbitals of the oxonium moiety. A central role of the antioxidant activity is the oxidation of anthocyanins’ phenolic hydroxyl groups; in particular, para- and ortho- phenolic groups are important for the formation of semiquinones and for the stabilization of one-electron oxidation products [15,16]. The 1,4 and the 1,2 conjugation are not the only electronic systems able to offer stabilizing condition for radicals on flavonoids. Anthocyanidins 3,5,7 and 3´and 4´ substituents, respectively on ring C, A and B are essential for the formation of different electronic delocalized and oxidized structures [15,16,17,18,19,20]. Figure 5 shows the possible antioxidant reaction mechanisms of cyanidin against a generic radical species (RO•).

Anthocyanins can quench reactive radical species by single electron transfer reaction and through hydrogen atom abstraction from phenolic groups. As shown in Figure 5, positions 3´ and 4´ are fundamental for the antioxidant capacity of these compounds. Catechol moieties (3´ and 4´ dihydroxyphenyl groups) on ring B are responsible for the antioxidant power of a wide range of polyphenols and flavonoids due to the ability to form ortho-semiquinones and subsequently ortho-quinones via two consecutive one electron transfer reactions [15,17,18,19]. In the case of cyanidin, the presence of additional hydroxyl groups in rings A and C can give rise to further centers of oxidant scavenge and radical delocalization. Position 3, 5 and 7 of cyanidin can be oxidized to form pseudo-semiquinone species, delocalizing electrons through the chromenylium cycle (rings A and C) and stabilizing the formed radicals. These species can be further oxidized to give rise to 3,5 or 3,7 pseudoquinonic structures, which can subsequently isomerize via keto-enol tautomerism [15,16,18].

**Figure 6:**
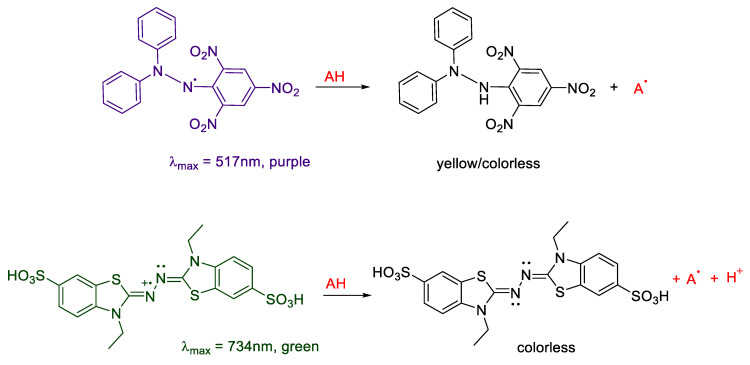
DPPH• hydrogen atom abstraction (HAT) and ABTS+• single electron transfer (SET) reactions with a generic antioxidants species (AH).

Several papers reported the antioxidant activities of anthocyanin extract from different agricultural and food matrices [20,21,22,23,24,25]. Most of these works describe the radical scavenging of anthocyanins by using different methods based on single electron transfer mechanisms (SET), hydrogen atom transfer (HAT) or by their combination [26,27]. DPPH• and ABTS+• antioxidant assays are two examples of SET and HAT direct electron transfer antioxidant assays (Figure 6).

Both these methods are based on the spectrophotometric decrease in absorbance of their quenched stable radicals. DPPH• shows a strong absorption maximum at 517 nm (purple). The color turns from purple to yellow followed by the formation of DPPH upon absorption of hydrogen from an antioxidant (e.g., flavonoids such as anthocyanins and anthocyanidins). ABTS+• is a stable radical cation, has a blue-green chromophore absorption and is produced by oxidation with potassium persulfate prior to the addition of antioxidants. The antioxidant activity of a wide number of natural products, including carotenoids, phenolic compounds, anthocyanins and other synthetic antioxidants, is determined by the bleaching of the ABTS+•, measured by the reduction in the radical cation as the percentage inhibition of absorbance at 734 nm.

Other utilized assays that demonstrated the strong antioxidant activity of anthocyanins and anthocyanidins are ferric reducing/antioxidant power (FRAP), deoxyribose assay (OH• radical scavenging) and NBT superoxide anion scavenging (O_2_−•) [17,21,22,23,24,25,26,27,28]. In all these assays, anthocyanin mixtures or pure isolated compounds revealed strong antioxidant activities. Interestingly, in most cases, the contribution of the anthocyanin fraction to the total antioxidant activity of a plant phenolic extract and matrices (i.e., red wine and blueberry) was prevalent with respect to the total crude extracts, indicating a high-impact biological and nutraceutical value of these compounds in plants and foods [17,20,24,25,29,30].

### 2.3. Extraction, Isolation and Chemical Characterization

There are different strategies for anthocyanins and anthocyanidin extraction from biological matrices based on their complexity and selective search for specific molecules with particular chemical features [31]. For example, the extraction of total anthocyanidins generally is performed by using aqueous/organic mixtures with a significant contribution of the organic part. Instead, the anthocyanins are often extracted with more hydrophilic solvents or with more prominent aqueous-based mixtures. In all this cases, it is very useful to maintain the ionization state of the compounds in the flavylium form, and this goal can be achieved by adding inorganic or organic acids to the aqueous phase. Plants, food and agricultural samples are usually extracted with ethanol/methanol: water mixtures (70-95:30-5) acidified with HCl, formic acid or other organic acids such as citric acid [32,33,34,35]. Another solvent commonly used for specific purposes is 70% aqueous acetone [36]. The use of strongest and more lipophilic organic solvents with the aid of ultra-sonication and temperature is chosen when the matrix is resistant to acidic homogenization, for example, in the case of plant seeds [36,37]. Liquid matrices instead (i.e., red wine, pomace and juices) are treated with alcoholic acidic solutions [38,39,40]. The extraction of anthocyanins using natural deep eutectic solvents (NADES) is a relatively novel, biocompatible and green approach at the forefront of sustainability, and has thus stimulated the interest of the scientific community in recent years [41]. Several methods were optimized for NADES-based anthocyanin extraction with the same efficacy of conventional organic solvent but with better yields compared to an exhaustive extraction with the organic solvent. NADES represent an excellent, useful updated strategy for green extraction and biocompatible preparation of anthocyanin-based products for pharmaceutical and nutraceutical applications [41,42,43,44].

After the extraction, depending on the matrices (fruits, leaves or liquid samples), the first step for purification procedures is to distinguish between green and red pigment-rich extracts. The first case indicates a strong chlorophyll component that must be preliminary purified, and the second one indicates a matrix richer in anthocyanin (fruits, flowers or liquid biological samples). After chlorophyll elimination, the purification is generally carried out by different chromatographic steps involving differential stationary phases on the basis of the purposes [31,45]. For aqueous extracts, a useful approach is the absorption of anthocyanins on solid phase extraction (SPE) resins such as C18 cartridges and on Sephadex matrices to eliminate more polar non-retained by-products. Further chromatographic steps generally involve normal phase silica gel stationary phases, reverse phase and cation exchange chromatography (i.e., Amberlite IRC 80, Amberlite XAD-7HP, and DOWEX 50WX8) [31,35].

Another very useful separation technique for anthocyanin purification is the high-speed counter-current chromatography (HSCCC) which allows the isolation of pure compounds to be used after XAD-7 resin column enrichment of crude extracts [46,47]. HSCCC biphasic systems composed of *n*-butanol/ethyl acetate/0.5% acetic acid (3:1:4) and 0.2% trifluoroacetic acid/*n*-butanol/tert-butyl methyl ether /acetonitrile (6:5:2:1) were observed to be an excellent mixture for chromatographic separation confirmed by HPLC and NMR analysis [46,48].

Pure compounds from different serial or parallel chromatographic separations (silica gel, cation exchange and/or revere-phase) can be structurally characterized by nuclear magnetic resonance (NMR), high-resolution and tandem mass spectrometry (HR-MS/MS^n^) and infrared spectroscopy (FT-IR). MS/MSn analyses are useful for the identification of specific fragmentation pathways [49,50,51,52]. For instance, anthocyanins can be differentiated/distinguished from their aglycons (anthocyanidins) by mass spectrometric-induced loss of sugar moiety after MS/MS fragmentation. The aglycones instead are sensitive to cross-ring cleavages especially on ring C, giving rise to different oxonium fragments [53]. Infrared spectroscopy (FT-IR) analyses allow one to identify functional groups, since each functional group absorbs radiation in a characteristic frequency of the infrared spectrum. Typical bands corresponding to skeletal stretching vibration of the aromatic rings and =C-O-C group of flavonoids (1072, 1516 and 1261 cm^−1^) are visible. The bands at 1711 cm^−1^ belong to the stretching vibration of C-O, and bands at 1072 cm^−1^ corresponding to bending vibration of C-O-C groups can indicate the presence of carbohydrates (in the case of anthocyanins instead of anthocyanidins) [54].

Even if MS/MS and FT-IR can help in anthocyanin identification (especially for known compounds referenced in the literature), NMR analysis remains the gold standard for the unequivocal elucidation of chemical structures of newly identified compounds [1,9,51,53,55,56,57,58]. An important NMR diagnostic signal for anthocyanin recognition and differentiation from other flavonoid skeletons is the presence in the proton ^1^H-NMR spectrum of the characteristic low field H-4 (8.6–9.1 ppm) singlet of flavylium salts. Aliphatic high field sugar signals (in the region between 3.5 and 5.5 ppm) of anthocyanins are a feature that characterizes glucosides from aglycones (anthocyanidins). The anomeric proton and carbon signals on ^1^H- and ^13^C-NMR spectra appear considerably downfield with respect to other sugar resonances. Large coupling or small coupling constants of the anomeric protons allows the assignment of respectively β or α sugar configuration for O- linked glycosides [55]. Finally, two-dimensional NMR (2-D NMR) spectra produced by homonuclear (^1^H-^1^H) and heteronuclear (^1^H-^13^C) experiments (i.e., TOCSY; HSQC; HMBC and ROESY/NOESY) allow for the complete structure elucidation of both aglycone and sugar parts of the molecules, and of the overall atoms space connectivity and stereochemistry, especially in structurally complex anthocyanidins and anthocyanin [55].

### 2.4. Analytical Methods

#### 2.4.1. Spectrophotometric Measurements

**Figure 7:**
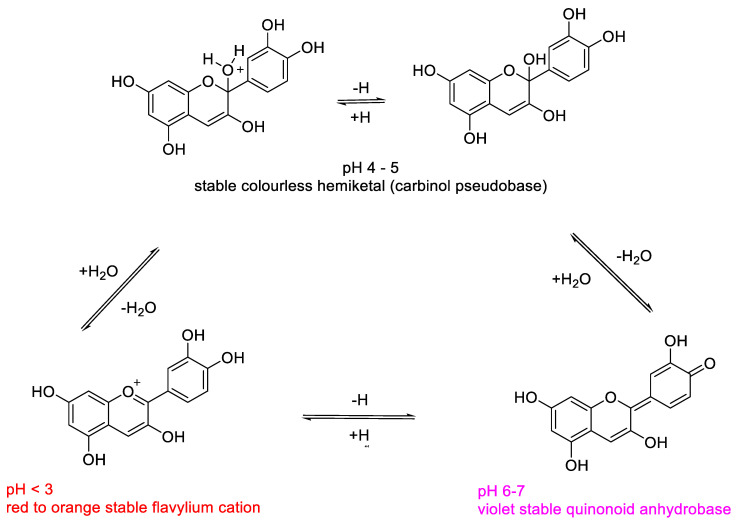
Stable colored and colorless forms of cyanidin at different pH values.

Several papers report the determination of anthocyanins in diverse biological samples such as plants extracts, food, and agricultural samples [9,31,32,34,49,59,60,61]. The spectrophotometric quantification of total monomeric anthocyanins is based on the characteristic light absorbance that these compounds possess depending on their ionization state [1]. The strong red-orange color of anthocyanins in acidic media is associated with their high molar extinction coefficient in the visible red light range between 500 and 550 nm (ε = 20,000–40,000) depending on the molecule [1,9,14]. As shown in Figure 4, there are different electronic delocalization states of the molecules in a different range of pH, each one related to a color. The majority of pH values are associated with characteristic absorptions due to the extension or decrease in the electronic conjugation between rings A-B and ring C across aromatic or quinonic intermediates. At very high pH values, the compounds lose their color as consequences of their degradation. However, there is another colorless range of pH which preserves the integrity of the molecules with no spectroscopic properties in the visible light range. At pH values between 4 and 5, anthocyanins are present in the hemiketal form (carbinol pseudobase), in which the conjugation and electronic delocalization among the three ring are disrupted (Figure 7).

**Figure 8:**
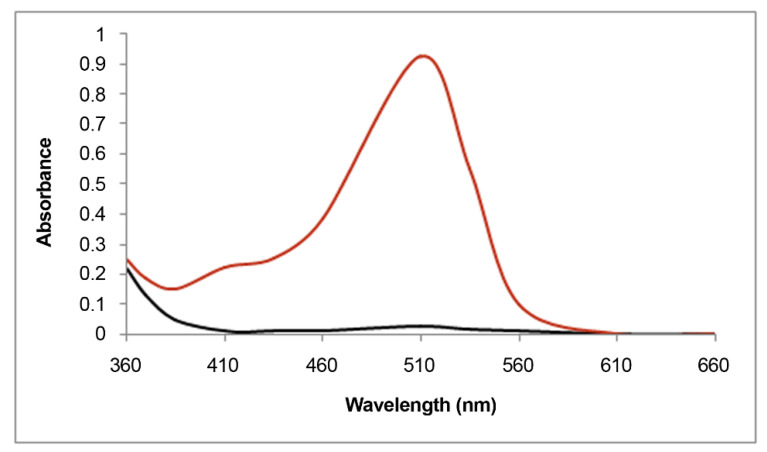
UV-visible absorption spectra of cyanidin-3-*O*-β-glucoside at pH 1.0 (red line) and pH 4.5 (black line).

The most reliable method for the spectroscopic quantification of anthocyanins is the pH-differential method in which UV-visible absorption spectra of the samples are recorded at pH 1.0 and 4.5 (Figure 8) [1,14,60,61,62,63,64,65]. The difference between the two λmax absorption values (in visible light range) allows accurate estimation of the total monomeric anthocyanins, even in the presence of other colored pigments and other interfering polymers. The general formula for anthocyanin quantification within the linear range of common spectrophotometers is as follows:

ΔAbs = (Abs λmax) pH 1.0 − (Abs λmax) pH 4.5

The quantification in complex biological extracts is possible by calculating the concentration of the predominant anthocyanin using the differential absorbance value (ΔAbs) molecular weight and extinction molar coefficient (MW and ε, respectively):

Anthocyanin [mg/mL] = (ΔAbs × MW × dilution factor)/ε

Usually, quantification of uncharacterized and unknown samples is performed using cyanidin 3-*O*-β-glucoside (chrysanthemin) as an equivalent for the estimation of total monomeric anthocyanins. In these case, MW = 449.2 and ε = 25740 (0.1 N aqueous HCl, λmax 520 nm) are used for the red pigment content calculation [14,66,67].

**Figure 9:**
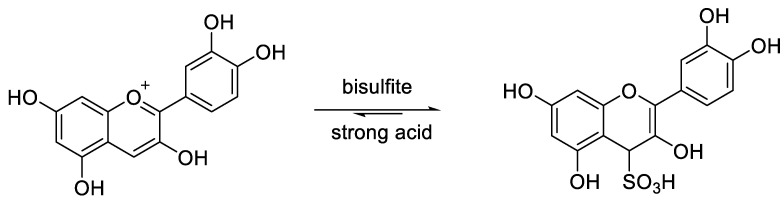
Flavylium cation form (**left**) and colorless cyanidin–sulfonic acid adduct after bleaching reaction with bisulfite (**right**).

Another step of this spectrophotometric analysis is the estimation of degraded and polymerized anthocyanins that could contribute to color intensity. For this purpose, the bisulfite reaction is the method used to evaluate the polymer contribution and determine the degradation index. Monomeric anthocyanins react in position 4 (electrophilic C-4) with bisulfite to give sulfonic acid adducts (Figure 9) which do not preserve the oxonium moiety and stop the ring electronic conjugation responsible for anthocyanin coloration [14,62,63,64].

The method implies the measurement of the absorbance values for samples treated and not with bisulfite to estimate respectively the polymeric color and total color density. The sample treated with bisulfite will develop a coloration that is the result of anthocyanin polymeric species that are resistant to bisulfite bleaching, while the untreated sample will produce a total color density resulting from all the pigments in the polymeric or monomeric form. The ratio between the two values gives an estimation of the color intensity contribution from the polymerized material [62,64].

#### 2.4.2. Chromatographic Analyses

Anthocyanin chromatographic analyses are mostly based on high performance and ultra-performance liquid chromatography (HPLC and UHPLC) methods coupled with spectrophotometric UV-visible and/or mass spectrometric detection [31,51]. The most used and useful HPLC and UHPLC methods for flavonoid analyses utilize reverse phase columns, in general C18 silica bounded stationary phases, of different diameters and particle size. Common 5 µm particle size HPLC columns are still wide spread in most of the analytical laboratories and still possess good selectivity and resolution of complex mixture of anthocyanidins and anthocyanins [40,57,59,60,68,69]. The upgrade to more powerful columns with smaller diameters and particle size (2.6 or 1.7 µm) is limited in many cases to the availability of UHPLC instruments and more modern chromatographic equipment. However, both methods still give acceptable separations of anthocyanins in rich and complex samples. The reverse-phase chromatographic analyses generally employ aqueous/organic gradients using acidified water and acetonitrile or methanol as organic solvent. The use of formic acid in the mobile phase is most suitable for subsequent mass spectrometric detection, as well as the use of low flow rates, which make the UHPLC system more suitable for the coupling with single quadrupole and triple quadrupole mass spectrometers [31,52,69]. The analysis of anthocyanins using HPLC-DAD (photodiode array detector) is probably the most used technique for the determination and the quantification of these compounds. Selective wavelengths selection (i.e., 520 nm) and the use of linear calibration curves (high purity analytical standards) allow the precise and reliable quantification of many compounds simultaneously. Several validated HPLC methods are reported in the literature with different modifications and with the use of different reverse phase columns for better separation of specific anthocyanins. Even if a lot of pure anthocyanins are commercially available as analytical standards for the quantification of known compounds, there are still several molecules not available and/or recently discovered with different modifications, especially in the region of the molecules involving sugar moieties. In these cases, UHPLC (or even HPLC) coupled with mass spectrometry (MS) is much more useful. MS detection methods possess many advantages for anthocyanin analysis [36,37,40,45,51,52,60,61,69,70,71]. First, anthocyanins are intrinsically charged compounds (oxonium charge stabilized in acidic conditions), making them ideal for HPLC/UHPLC-MS and absolutely compatible with acidic modifiers used in common mobile phases. Another advantage of mass spectrometric detection is the possibility to monitor single molecular ions in the same chromatographic course (SIR/SIM, single ion recognition/monitoring) that provides the possibility to separate ions in a second dimension (the mass/charge ratio) and quantify co-eluting peaks in the chromatographic analysis (excluding isobaric species). Another advantage is the possibility to use combined parameters, such as retention behavior, UV-visible spectral data and molecular mass, to identify unknown compounds in the sample and of which no analytical standards are commercially available. For this purpose, tandem mass spectrometry (MS/MS^n^) coupled with HPLC or UHPLC is a powerful tool. MS/MS^n^ gives the possibility to analyze different molecular transitions and fragmentations and to compare them with the data present in the literature and with most up-to-date mass spectrometric data banks.

## 3. Anthocyanins in Food

### 3.1. Natural Sources of Anthocyanins

Anthocyanins are widespread in red/blue fruits and vegetables and their content in plants varies markedly among different species, depending on cultivar or variety, growing area, climate, farming methods, harvest time, ripening, seasonal variability, processing and storage, temperature and light exposure. Berries such as strawberries, blueberries, blackberries, blackcurrant, redcurrant and raspberries are a rich source of anthocyanins, with levels ranging from about 100 to about 700 mg/100 g of fresh product [10,72,73], but the highest content is found in elderberries and chokeberries, which can contain up to 1,4-1,8 g of anthocyanins per 100 g of product [10,72]. Other good sources of anthocyanins include purple corn, cherries, plums, pomegranate, eggplant, wine, grapes, and red/purple vegetables such as black carrots, red cabbage and purple cauliflower which may contain from a few milligrams up to 200–300 mg/100 g of product [72,73]. More recently, anthocyanins have been identified in numerous berries whose production and consumption is steadily increasing, such as maqui [74,75], myrtle [76,77], and açai [78,79,80].

Cyanidin, having two hydroxyl groups on the B-ring, is the most widely distributed pigment among plants. The most represented anthocyanin in edible plants is cyanidin-3-*O*-β-glucoside, followed by delphinidin, pelargonidin and peonidin glucosides [6]. In general, hydroxylation causes a blue hue and reduces stability, whereas methylation induces red hue and improves stability. The hydroxyl groups may be modified by glycosylation or acylation. Both modifications affect the physical and chemical properties of anthocyanins and modify the chemical reactivity and polarity of the molecules [7].

Fruits are the most common dietary source of anthocyanins, providing up to 70% of daily intake, predominantly from apples, pears, berries, stone fruits and grapes. Wine may contribute up to 25% of intake across Europe [81]. In the US and Northern Europe, the main dietary sources are berries [82,83]. Anthocyanins are not considered as essential nutrients hence no recommended daily intake has been established, however China has recently suggested a daily intake of 50 mg [84]. Though the evaluation of anthocyanin daily intake is cumbersome and inaccurate, mainly due to the incomplete data on the anthocyanin quantities in food, it has been estimated that the daily intake is about 12.5 mg/day in the US [72], while in Europe mean intake ranges from 19 to 65 mg/day for men and from 18 to 44 mg/day for women [81]. An Australian study reports that the mean anthocyanin intake is about 24 mg/day [85], whereas in Finland, the daily intake has been estimated to be up to 150 mg/day [83], with the primary source being the consumption of berries.

Given the health-protecting effects of anthocyanins, promoting the intake of fresh fruits and vegetables may be desirable to guarantee an adequate level of antioxidant and protecting substances at the plasma level. Indeed, a regular intake of fruits and vegetables is an important factor of a healthy lifestyle and can provide protection against chronic and degenerative diseases. For instance, the adherence to Mediterranean diet, which is rich in food containing anthocyanins (fruits, berries, vegetables, beans and cereals), has been associated with a reduction in inflammation markers and a lower risk of various diseases, including obesity, diabetes, cancer, and cardiovascular disease [86]. Conversely, low intake of fruits and vegetables accounts for an estimated 1.7 million deaths globally, including but not limited to those caused by gastrointestinal cancer (14%), ischemic heart disease (11%), and stroke (9%).

### 3.2. Anthocyanins as Natural Food and Beverages Colorants

The food industry uses many chemical substances as food colorants. However, this use poses a number of problems, mainly due to health risks. Indeed, synthetic dyes have been suspected to cause adverse behavioral and neurological effects [87]. Anthocyanins, being safe and potentially health protective, represent an attractive alternative to synthetic substances. Indeed, the use of anthocyanins as food colorants in foods and beverages is widely permitted within Europe (E163), Japan, the United States, and many other countries [7].

Products which could benefit from anthocyanin addition include soft drinks, syrups, jams, jellies, sweets, bakery or dairy products, and powders. Besides providing color to the food, anthocyanins can provide an additional double advantage. They can act as antioxidants protecting the food to which they are added, but they can also supply a distinctive quality to food as they can increase the nutritional potential, exerting health-promoting effects for consumers. However, the use of anthocyanins as natural food colorants poses several problems. Firstly, their stability is not optimal, as they tend to quickly degrade mainly due to light, oxygen, enzymes, metals, presence of other oxidants, pH and temperature [6,88,89,90,91]. Secondly, they are quite expensive compared to synthetic molecules. Thirdly, they could cause off-flavors in food products, as in the case of anthocyanins extracted from red radish [92].

Various methods and techniques have been applied to improve anthocyanins’ stability, including microencapsulation, oxygen exclusion, co-pigment addition with colorless molecules in solution and chemical derivatization such as acylation. Microencapsulation or complexation with biopolymers seem to provide an efficient protection, particularly when maltodextrins and beta-glucans are used [93,94,95,96]. Microcarriers may be produced by spray-drying or freeze-drying. An alternative approach to microencapsulation is represented by nanoformulations such as nanoliposomes or nanoemulsions [97].

Sources of anthocyanins as potential food colorants are grape skin, radishes, red potatoes, red cabbage, and purple sweet potatoes [98], black carrots [99], black beans [100], chokeberry [101], *Thymus moroderi* [102], prunes [103], *Hibiscus sabdariffa* [104] and many others. Generally, acylated anthocyanins are preferred as food colorants as they are more stable than nonacylated anthocyanins, though some fruits such as elderberry and chokeberry can be used to extract high amounts of nonacylated anthocyanins at low cost, thus, they also have potential use in the food industry [7].

### 3.3. Bioavailability of Anthocyanins

It is generally recognized that to exert a biological effect a substance should be absorbed and reach tissues in amount high enough to elicit a response, i.e., it must be bioavailable. When ingested, even in high amounts, anthocyanins rarely reach a concentration in plasma that could be considered therapeutically active. However, numerous epidemiological and experimental data indicate that these compounds have many beneficial effects on health. Apparently, anthocyanins have an extremely low bioavailability and reach only trace concentrations in plasma, but their metabolism and distribution could greatly affect the amount determined at the plasma level. Anthocyanidin structure is the predominant factor governing the absorption of anthocyanins. Indeed, pelargonidin-based anthocyanins are more readily absorbed than anthocyanins with more substituents on the B-ring [105].

Anthocyanins are absorbed in the gut and reach the liver via the portal vein. Here, they are metabolized, secreted and reabsorbed in the enterohepatic circle to restart the pathway all along [106]. In this manner, the anthocyanins become more and more metabolized, giving rise to many different molecular intermediates that may possess specific properties and biological activities. After absorption, anthocyanins are metabolized by phase I and phase II enzymes, which generate hydroxylated, glucuronidated, sulfated, and methylated molecules mainly in the liver, but also at the renal and enterocyte level [106].

Anthocyanins may be metabolized all along the gastrointestinal tract. In the mouth, anthocyanins are mostly metabolized by oral microbiota which can remove glycosidic groups and transform anthocyanins into the corresponding chalcones [106]. In the stomach, anthocyanins are rapidly absorbed [107,108,109], but the site of maximal absorption is the gut.

An enzyme present on the brush border of the enterocytes, the lactase phlorizin hydrolase, releases the aglycone that may then enter the epithelial cells by passive diffusion as a result of its increased lipophilicity and its proximity to the enterocyte membrane. β-glucosidases also liberate free aglycones which are more hydrophobic and have a lower molecular weight of the corresponding glycosides, hence, they are more membrane permeable. Glycosides also are absorbed by the small intestine, possibly by the sodium-dependent glucose transporter SGLT1. Acylated anthocyanins also are absorbed, but the amount is 4-fold lower than that of non-acylated anthocyanins [44,93].

Unabsorbed anthocyanins reach the colon where they are extensively modified by the colonic microflora which may releases aglycones and generate simple phenolics [110] that then may be absorbed by the colonic mucosa. Theoretically, the absorption process at the colonic level is much less efficient than that at the small intestine level, however, a recent study by Mueller et al. [111] demonstrated that in ileostomized patients the amount of anthocyanins absorbed is significantly lower than that of patients with intact intestine, thus pointing to a major role of colon in the absorption of these compounds. The extensive metabolism exerted by the microflora in the colon plays a critical role in the bioavailability of anthocyanins. Bacteria of the intestinal microbiota have a vast array of enzymes which participate in metabolism of anthocyanins, including β-d-glucosidase, β-d-glucuronidase, α-galactosidase and α-rhamnosidase activities, which lead to the cleavage of glycosidic bonds. Bacterial metabolism of anthocyanins leads also to the breakdown of the heterocycle, thus generating simple phenolics. Hence, anthocyanins can be transformed into phenolic acids such as gallic, protocatechuic, syringic, *p*-coumaric, vanillic, cinnamic, phenylpropionic or homovanillic acid, or into simple phenolics such as hydroxytyrosol or benzaldehydes [112]. Particularly, cyanidin-glucosides are transformed into protocatechuic acid [113], whereas malvidin, pelargonidin, delphinidin and peonidin are metabolized to syringic, 4-hydroxybenzoic, gallic and vanillic acids, respectively [114,115]. In this way, anthocyanins are transformed into more bioavailable and more readily absorbable forms.

It is of great interest to note that these products may in turn modulate the colonic microbiota composition. Indeed, many authors observed that the intake of anthocyanins causes an increase in beneficial bacteria such as Bifidobacteria, Lactobacilli or Actinobacteria [116,117,118]. It is well-known that probiotic bacteria may exert numerous beneficial effects on human health, hence the positive effects observed following intake of anthocyanins could in part be due to the modulation of intestinal microbiota.

One of the mechanisms by which anthocyanins may increase the amount of probiotic bacteria in the gut is the production of short chain fatty acids (SCFA). The metabolism of anthocyanins by intestinal bacteria produces the breakdown of glycosidic bonds and in turn the production of SCFA and phenolic acids, which both induce a decrease in pH and generate a milieu that stimulates the proliferation of probiotic bacteria [116].

Bioavailability may also be affected by the food matrix and the food processing, both of which may modulate anthocyanin bioaccessibility. Meal composition could also be a factor affecting bioavailability, i.e., food components like alcohol, sugars, proteins and fat may modulate intestinal absorption [119]. Interestingly, thermal processing can have a double effect on bioavailability: on the one hand, it may decrease anthocyanin content due to thermal instability of the pigments, on the other hand, it may increase anthocyanin bioaccessibility by partially degrading food matrix [119].

Bioavailability can be affected by the microencapsulation or nanoformulation of anthocyanins [97]. It has been hypothesized that besides providing improved stability, encapsulation may favor intestinal absorption and metabolism of anthocyanins. Indeed, in vitro studies demonstrated that nanocomplexes with chitosan hydrochloride, carboxymethyl chitosan and β-lactoglobulin may favor the bioavailability of anthocyanins in a simulated gastrointestinal tract [120]; that bilberry anthocyanins encapsulated in liposomal micelles were more bioavailable at the cellular level in vitro; and that their efficacy as anticancer agents was significantly higher than that of non-encapsulated anthocyanins [121]. However, a recent paper by Mueller et al. [122], in which the anthocyanins were encapsulated in whey proteins, demonstrated that this formulation had little effect on the stabilization of anthocyanins during intestinal passage, and increased the rate of degradation when compared to non-encapsulated anthocyanins. The authors hypothesized that whey protein encapsulation might increase systemic concentrations and short-term bioavailability by prolonging the duration of stomach passage, leading to higher concentrations of anthocyanins and their degradation products in urine.

The different results obtained in vivo and in vitro may be due to the different type of nanoformulation or encapsulation of anthocyanins, or to the body response to encapsulated anthocyanins, which may be absorbed more rapidly and hence more rapidly metabolized by the human organism.

## 4. Biosynthesis of Anthocyanins and Gene Expression

### 4.1. Biosynthetic Pathway

**Figure 10:**
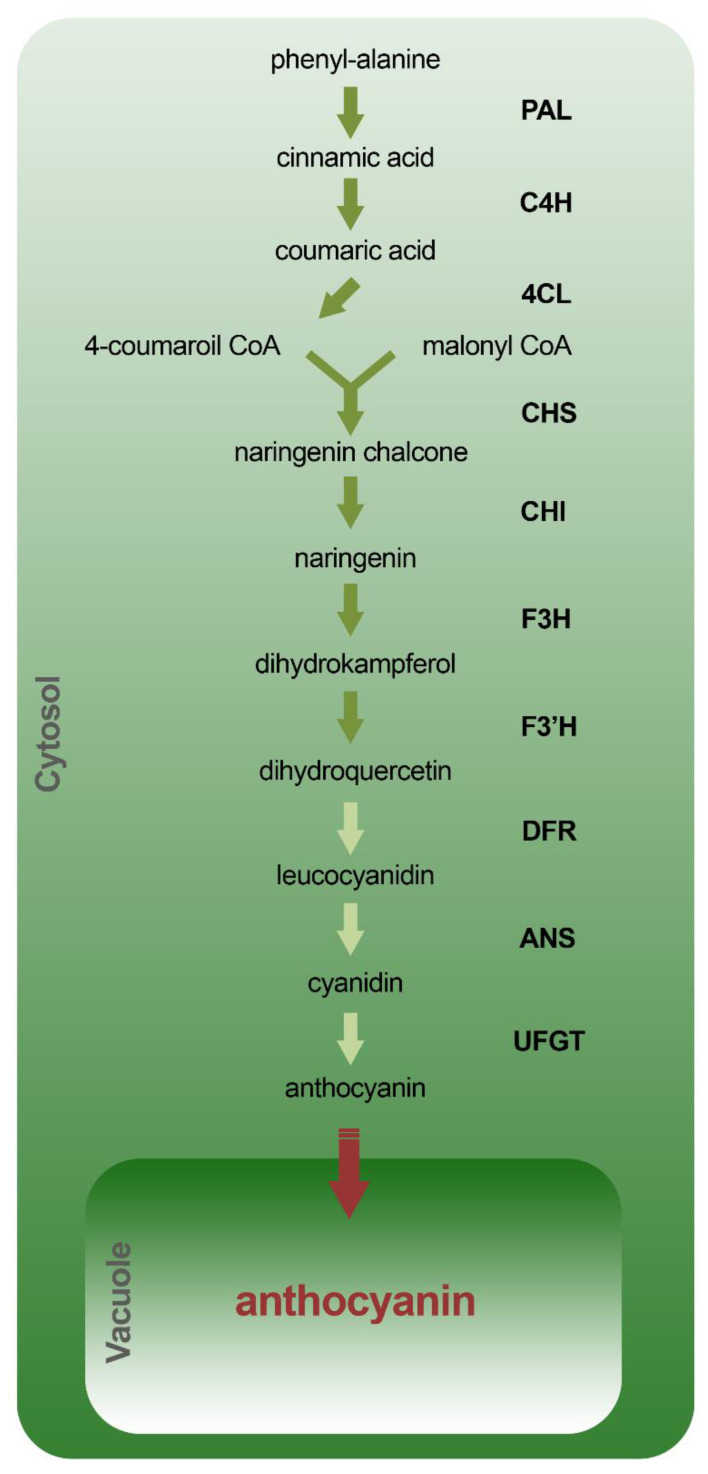
Schematic representation of anthocyanin biosynthetic pathway. PAL, phenylalanine ammonia-lyase; C4H, cinnamate-4-hydroxylase; 4CL, 4-coumaroil CoA ligase; CHS, chalcone synthase; CHI, chalcone isomerase; F3H, flavanone 3-hydroxylase; F3′H, flavonoid 3′-hydroxylase; DFR, dihydroflavonol reductase; ANS, anthocyanidin synthase; UFGT, UDP-glucose:flavonoid-3-*O*-glycosyltransferase.

The biosynthetic pathway of anthocyanins has been well characterized both in an *Arabidopsis thaliana* model plant and in various crops, and appears to be strongly conserved. The biosynthetic pathway of anthocyanins constitutes an important branch of the phenylpropanoid pathway and shares, in the initial stages, some biosynthetic enzymes for other flavonoids such as flavones and flavonones. It starts with phenylalanine which is converted into cinnamic acid by phenylalanine ammonia-lyase (PAL) (Figure 10). The cinnamic acid is then converted into coumaric acid by the action of cinnamate-4-hydroxylase (C4H) and subsequently converted into 4-coumaroil CoA by the 4-coumaroil CoA ligase (4CL). Following condensation of 4-coumaroil CoA with malonyl CoA, naringenin chalcone is produced by chalcone synthase (CHS) and subsequently converted into naringenin by chalcone isomerase (CHI) and dihydroflavonols, such as dihydrokaempferol and dihydroquercetin from flavanone 3-hydroxylase (F3H) and the flavonoid 3′-hydroxylase (F3′H), respectively. The last steps of the biosynthetic pathway lead to the production of leucocyanidins, cyanidins and anthocyanins by dihydroflavonol reductase (DFR), anthocyanidin synthase (ANS) and UDP-glucose:flavonoid-3-*O*-glycosyltransferase (UFGT), respectively. While the biosynthesis of anthocyanins occurs in the cytosol, they are stored in the vacuole by specific transporters. In Arabidopsis for example, the TT12 and AHA10 genes have been associated with the transport and vacuolar accumulation of anthocyanins in the seed [123,124]. In particular TT12, which encodes for a membrane protein belonging to the “multidrug and toxic efflux antiporter” family, has been shown to be involved in the accumulation, at the vacuolar level, of glycosylated flavan-3-ols and protoanthocyanidins [123,125]. AHA10, which encodes for a plasma membrane H+-ATPase, is instead responsible for the acidification of the vacuole [124]. tt12 mutants as well as aha10 mutants, show comparable phenotypes, characterized by seeds with transparent heads and alterations in the accumulation of anthocyanins inside the vacuole. Precisely for this reason, it has been hypothesized that AHA10 can provide the proton gradient necessary for the mediated TT12 transport [124]. Moreover, it has recently been demonstrated that “ATP-binding cassette (ABC)” and “H+” antiport, also favored by the close interaction between anthocyanins and GSH, are involved in the vacuolar transport of these molecules in Arabidopsis vegetative tissues [126].

### 4.2. Modulation of the Enzymatic Synthesis

#### 4.2.1. Synthesis Regulation

The regulation of anthocyanin levels during development or in response to environmental stimuli occurs mainly by regulating the gene expression of biosynthetic genes. This regulation can take place at different levels: epigenetic, transcriptional, post-transcriptional and post-translational [127,128]. Although the regulation of biosynthetic genes of anthocyanins has been more investigated at the transcriptional and post-transcriptional level, recent evidence has also highlighted a role of the epigenetic regulation. Cai and colleagues [127] studied the genetic interactions between the tri-methylation of lysine 4 on histone H3 (H3K4me3) and the SWR1 chromatin remodeling complex in the control of anthocyanin biosynthesis. A variant of histone H2 (H2A.Z) negatively regulates the accumulation of anthocyanins by repressing the expression of biosynthetic genes in *Arabidopsis thaliana*. In particular, the authors shown that H3K4me3 in the biosynthetic genes of anthocyanins is negatively associated with the presence of H2A.Z, suggesting its antagonistic role. In fact, in mutants deficient in the replacement of histone H2A.Z, the increase in the amount of anthocyanins is associated with the increase in H3K4me3. At the transcriptional level, the expression of the anthocyanin biosynthetic genes are regulated by R2R3-MYB transcriptional factors such as MYB11, MYB12 and MYB111 [129], and by a ternary complex known as the MBW complex, formed by R2R3-MYB, bHLH and WD40 factors [130]. MYB75 is a transcription factor belonging to the R2R3-MYB family, which is part of this complex [131]. Arabidopsis plants overexpressing the MYB75 gene, for example, show a greater accumulation of anthocyanins in roots, stems, leaves and flowers, while, on the contrary, myb75 mutants show a lower accumulation of these molecules [132,133,134]. The importance of MYB75 in regulating the accumulation of anthocyanins would seem to be confirmed also in some crops such as in *Actinidia chinensis* plants (kiwi) [135]. In particular, AcMYB75 shows an expression pattern linked to the accumulation of anthocyanins during fruit development, localizes in the nucleus and binds, both in the hybrid yeast system and in vivo, specifically the promoter of the ANS gene. Furthermore, in Arabidopsis 35S::AcMYB75 plants, the expression of some biosynthetic genes is strongly altered and there is a significant accumulation of anthocyanins in leaves. Other transcription factors of the ternary complex and belonging to the R2R3-MYB family are, for example: MYB90, MYB113 and MYB114 [136]. The factors of the ternary complex belonging to the bHLH family, GLABRA3 (GL3), ENHANCER OF GLABRA3 (EGL3) and TRANSPARENT TESTA 8 (TT8), play an important role and seem to have specific roles during development or in response to environmental stimuli [137]. Finally, regarding to the transcription factors belonging to the WD-40 family, only TRANSPARENT TESTA GLABRA 1 (TTG1) has been characterized and associated with the regulation of anthocyanin biosynthesis. TTG1 encodes a 341 amino acid protein that has four WD-40 repeats, is expressed during all stages of plant development and is present in all tissues [138]. Furthermore, it does not change its expression in response to environmental stimuli [139]. For these characteristics, ttg1 mutants show pleiotropic effects such as alteration of the development of trichomes; alterations in the accumulation of anthocyanins, causing a transparent head phenotype in the seed coat; and altered development of the root hairs [138,140,141]. In general, the whole ternary complex functions as a transcription activator of the anthocyanin biosynthetic genes. However, the presence of repressors can quell the transcription of these genes both through a direct link to the promoters, such as MYB7 and MYB4 belonging to the R2R3-MYB family, and through the inhibition of factors of the ternary complex formation, such as CAPRICE (CPC) and MYBL2 belonging to the R3-MYB family [142,143,144]. In particular, it has been shown that AtMYB7 and AtMYB4 are able to inhibit the synthesis of anthocyanins through transcriptional repression of the DFR and UGT genes. Indeed, atmyb7 and atmyb4 mutants show an increased expression of these genes and consequently an increase in anthocyanin content [142]. Some transcription factors can interfere with the formation of the MBW complex by contrasting the link between the several factors of complex; this is the case, for example, of SQUAMOSA-PROMOTER BINDING PROTEIN-LIKE (SPL) which hinders the connection of the MYB factors with the other elements of the complex [145]. It has been shown that plants overexpressing the CPC gene show an altered expression of seven genes involved in anthocyanin biosynthesis through direct competition with transcription factors such as MYB75 and MYB90 [143]. A similar mechanism has also been demonstrated for MYBL2 [144]. In addition, MYBL2 contains a C2 motif capable of directly repressing the transcription of positive transcription factors such as TT8 and MYB75 [146].

Compared to transcriptional regulation mechanisms, post-transcriptional and post-translational ones are less known. However, today, we know that several microRNAs can regulate the expression at a post-transcriptional level of factors such as MYBL2, SPL, MYB75. In particular, miR156, a microRNA involved in numerous developmental processes, binds the mRNA of SPL by regulating its expression at a post-transcriptional level [145]. Similarly, miR858a represses MYBL2, leading to the activation of the biosynthetic pathway of anthocyanins [147]. The mRNAs of factors MYB75, MYB90 and MYB113 were found to be the target of miR828 conserved in both mono- and di-cotyledons and constitutively expressed in *Arabidopsis thaliana*. The transcriptional levels of MYB75, MYB90 and MYB113 are strongly repressed in 35S::miR828 plants which consequently show reduced transcriptional levels of the PAL, CHS, CHI, F3H, F3’H, DFR genes and reduced levels of anthocyanin accumulation [148]. Light is one of the environmental factors that most influences the development of plants. One of the answers, following exposure of a plant to light, is the synthesis and accumulation of anthocyanins which are therefore absent in dark conditions. One of the mechanisms that regulates this process is at post-translational level by regulating, for example, directly the activity of MYB75 by MAP KINASE 4 (MPK4) or through its degradation via proteasome [149]. In particular, it has been shown that under light conditions, MYB75 is phosphorylated by MPK4; this phosphorylation increases the stability of MYB75 and consequently its activity. On the contrary, in dark conditions, MYB75 and MYB90 are degraded via proteasome by the CONSTITUTIVELY PHOTOMORPHOGENIC1 / SUPPRESSOR OF PHYA-105 (COP1 / SPA) complex [150]. Indeed, cop1 and spa mutants accumulate anthocyanins both in light and dark conditions.

#### 4.2.2. The Role of Synthesis Regulation during Development and Environmental Responses

As previously observed, the anthocyanin synthesis in plants is associated with the presence of light. Their quantity also varies according to the species and variety of the plant, its stage of development and according to the organ or tissue that is taken into consideration. The main function of the anthocyanins, present in the flowers or in the epidermis of the fruits, is to attract animals and pollinating insects to easily disseminate the seeds or to facilitate the spread of pollen. It is therefore not surprising that the synthesis of these molecules is concentrated in these structures and during their development. Jaakola and colleagues [151], for example, studied the variation of the expression of the biosynthetic genes of anthocyanins by relating them to the accumulation of these during the development and maturation of the fruits of the blueberry plant. In the early stages of development, the main molecules present were procyanidins and quercetin. However, during development, the levels of these molecules drastically decreased, while the levels of anthocyanins increased. Parallel to the accumulation of anthocyanins inside the blueberries, the expression of the main biosynthetic genes of the anthocyanins (PAL, CHS, F3H, DFR, ANS) increased and then dropped at the end of maturation. Furthermore, no expression of these genes was observed in the white and pink mutants. Similar results have been obtained in the snapdragon (*Antirrhinum majus*) [152], petunia (*Petunia hybrida*) [153], pea (*Pisum sativum*) [154], and strawberry (*Fragraria ananassa*) [155].

In plants, sessile organisms have developed a series of biochemical and molecular integrations that allow them to react to adverse conditions. An important role of anthocyanins within plants is to provide them with adequate protection against environmental factors that could seriously endanger their survival. It is known that under stress conditions, such as low temperatures, water or saline stresses, conditions of high luminous fluence, the metabolism and the quantity of anthocyanin changes. Interestingly, it was observed that different types of stress induce the synthesis of different types of anthocyanins, suggesting a different physiological role within the plant [156]. Crifò and colleagues [157] studied anthocyanin accumulation and gene expression of related genes in blood oranges under low storage temperature conditions. In particular, they observed an increase in anthocyanin levels and in the expression of related genes in conditions of low temperatures (4 °C) when compared with controls at 25 °C. Similar results were obtained by He et al. [158] who investigated the effect of low temperatures on anthocyanin accumulation and expression of related genes in seedling of purple head Chinese cabbage, white head parent Chinese cabbage, and its purple male parent. The purple cultivars showed a strong accumulation of anthocyanins at low temperatures compared both to the controls grown at 25 °C and to the white cultivars. Interestingly, the biosynthesis genes and transcription factors BrMYB2 and BrTT8 were found to be overexpressed, while most of the genes of the phenylpropanoid pathway were found to be downregulated. Furthermore, the negative regulators BrMYBL2.1 and BrLBD38.2 in the white variety were found to be overexpressed. In contrast, it appears that high temperatures lead to a decrease in anthocyanin levels and a down-regulation of the related biosynthetic genes [155,159]. Anthocyanin biosynthesis is strongly up-regulated in conditions of saline stress [160] or in conditions of water scarcity [161]. According to a study carried out on grape berries grown in drought conditions, in field conditions and for two consecutive years, 84% of the total variation in anthocyanin content was explained by a linear relationship of the mRNA accumulation of the UFGT genes, CHS, F3H. Furthermore, the concomitant induction of genes related to brassinosteroids, plant hormones that are important for fruit development and maturation, suggests an inter-relationship between the development signaling pathway and the environmental signaling pathway [161]. Although the molecular mechanism underlying the increase in anthocyanin levels in response to water stress is not yet fully understood, many steps forward have recently been made. Indeed, recent studies suggest that the accumulation of high levels of anthocyanins, in plants under drought stress, may be linked to the regulation of miR156 through the increase in abscisic acid (ABA) level, the main plant hormone that responds to several environmental stresses [162]. Furthermore, it would appear that moderate levels of miR156 overexpression in alpha-alpha plants suppress the SPL13 expression and increase the expression of WD40-1 and consequently of DFR [163]. Finally, the modulation of anthocyanin levels in plants also plays an important role in the responses against light and/or UV-B ray stresses [164].

#### 4.2.3. Biotechnological Approaches to Increase Anthocyanin Levels in Food

Though anthocyanins are molecules widely present within the plant kingdom, their quantity in foods which we usually consume is modest. However, a growing series of studies are highlighting the beneficial effects of these molecules on human health and their protective role against several chronic and degenerative diseases. For this reason, many research groups are concentrating their efforts, not only to better understand the molecular mechanisms that underlie the synthesis of these molecules but also to increase their levels into the plants that we consume on a daily basis. Narrowing our focus to the *Solanum lycopersicum* plant (tomato), one of the most widely used plant in the world, important results have been obtained both by classic genetic approaches and by modern genetic engineering techniques. The cultivars produced through classical genetics and conventional breeding were obtained by crossing commercial varieties with wild-type plants capable of producing high levels of anthocyanins. For example, the dominant Aft allele and the recessive atv allele were introduced into *Solanum lycopersicum* from *Solanum chilense* and *Solanum cheesmaniae*, producing the Indigo Rose cultivar (“Sun Black”), characterized by a high amount of anthocyanins but limited to the fruit skin [165]. Better results have been obtained by applying genetic engineering techniques. The results obtained by Butelli and colleagues [166], who engineered tomato with two specific transcription factors (Delia (Del) and Rosea 1 (Ros1)) from snapdragon, must be highlighted. In this case, both skin and pulp of berries are characterized by an intense purple color. In pilot experiments, Trp53-/- mice susceptible to cancer, fed with a diet supplemented with purple tomato powder, showed a significant increase in life span compared to standard or supplemented with red tomato powder diet. Similar results, more recently, have been obtained by Sun and colleagues [167] in the Indigo Rose cultivar (previously mentioned). In particular, it has been shown that the Aft locus encodes for a functional anthocyanin biosynthesis activator called SIAN2-like, while atv encodes for a non-functional version of the SIMYBATV repressor. Furthermore, it has been shown that SIAN2-like is able to activate both the biosynthetic genes of anthocyanins and their regulatory genes, suggesting that SIAN2-like works as master regulator. Finally, it has been shown that the cultivated tomato varieties contain a non-functional SIAN2-like allele and are therefore unable to accumulate anthocyanins. Consistently, the expression of a functional SIAN2-like gene, under the control of the fruit-specific promoter SIE8, leads to the activation of the entire synthesis pathway, allowing the accumulation of anthocyanins not only in the skin but also in the fruit pulp.

## 5. Anthocyanins’ Health Effects on Cardiovascular and Neurodegenerative Diseases

This section focuses on providing scientific evidence from animal and human clinical studies to describe the impact of anthocyanins and microbial-driven anthocyanin on cardiovascular and neurodegenerative diseases. It is urgent to find preventive intervention strategies to slow down these age-related diseases progression. Cardiovascular diseases (CVDs) are the principal cause of morbidity and mortality worldwide, and deaths caused by neurodegenerative diseases more than doubled in the past six years, moving from the 14th to the 5th position in the global causes of deaths list between 2016 and 2020 [168].

### 5.1. Cardiovascular Diseases

#### 5.1.1. In Vivo

One of the common primary mechanisms of CVDs initiation and progression is chronic inflammation, particularly that affecting the endothelium. The permanence of inflammation in the walls of medium and large arteries promotes the initiation and progression of atherosclerosis, which is related to other forms of CVDs, such as hypertension, peripheral arterial disease, coronary artery disease, and ischemic stroke [169].

**Figure 11:**
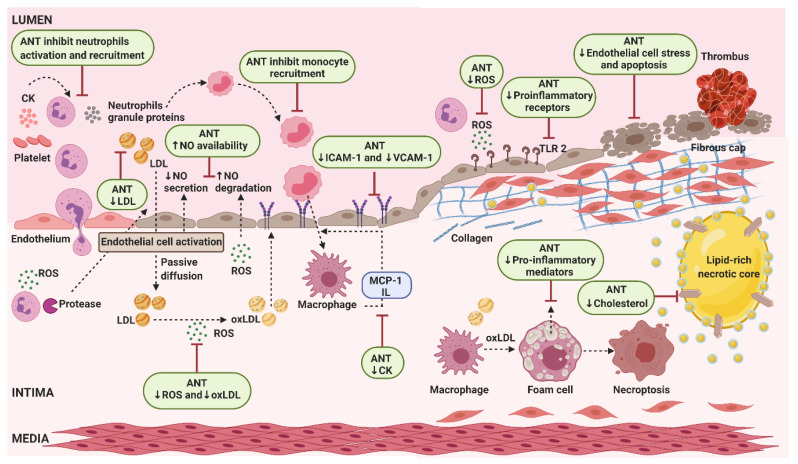
Anthocyanins’ protective effects against atherosclerosis. Anthocyanins’ (ANT) protection occurs in all atherosclerotic stages. ANT decrease plasma low-density lipoprotein (LDL), leading to a reduction in their accumulation in the walls of medium and large arteries. Therefore, ANT indirectly inhibit endothelial cell dysfunction/activation promoted by LDL. Endothelium damage impairs the release of nitric oxide (NO), which together with a local enhanced degradation of NO by increased generation of reactive oxygen species (ROS), decreases NO availability. ANT can increase NO availability by several mechanisms. After activation, endothelia start to express cell adhesion molecules on their surface (ICAM-1, intercellular adhesion molecule-1 and VCAM-1, vascular cell adhesion molecule-1) in order to recruit circulating monocytes to the site of oxidized LDL (oxLDL) accumulation. The expression of these adhesion molecules is downregulated by ANT. In the luminal side, ANT decrease chemokines (CK), which also results in a decline in myeloid cell recruitment. ANT counteract ROS in both the luminal and intimal side, reducing LDL oxidation in vessel wall. During atherogenesis progression, neutrophil-derived granule proteins stimulate macrophage activation to a proinflammatory state which can be inhibited by ANT. Both antioxidant and anti-inflammatory effects of ANT decrease foam cell formation. Moreover, ANT decrease cholesterol by reducing their accumulation in the lipid-rich necrotic core. During the late stages of atherosclerosis, ANT reduce the expression of Toll-like receptor 2 (TLR2) signaling in endothelial cells that regulate neutrophil stimulation of endothelial cell stress and apoptosis. The arrowhead denotes the routes of atherosclerosis progression, whereas the hammerhead represents the effects of ANT.

The apolipoprotein (apo)E-deficient mouse model develops marked hypercholesterolemia and spontaneous atherosclerosis and, therefore, is one of the models most used for exploring the effects of anthocyanins in any stage of this pathologic condition [170]. The main results of which are discussed in this paragraph and illustrated in Figure 11. An initial stage in atherosclerotic lesion development is endothelial cell activation caused by the accumulation of low-density lipoprotein (LDL) and other apoB-containing lipoproteins in the walls of large and medium arteries. Cyanidin-3-*O*-β-glucoside increases endothelial nitric oxide synthase phosphorylation and preserves nitric oxide availability [171], which promotes endothelial cell migration and survival [172,173]. Moreover, cyanidin-3-*O*-β-glucoside improves both the loss of endothelial progenitor cells function and endothelial repair, deaccelerating the atherogenesis caused by diabetes induced in apoE-deficient mice [174]. Activated endothelial cells release inflammatory mediators (e.g., MCP-1, monocyte chemoattractant protein-1) into the bloodstream, and start to express cell adhesion molecules on their surface (ICAM-1, intercellular adhesion molecule-1 and VCAM-1, vascular cell adhesion molecule-1), in order to attract circulating monocytes and other immune cells to the oxidized LDL (oxLDL) accumulation site [172,173]. Anthocyanin-rich extract from purple sweet potato and from red Chinese cabbage promotes a decrease in VCAM-1 level on plasma and in adhesion molecule expression on the arterial endothelium surface, respectively [175,176]. Protocatechuic acid (PCA) is the main human metabolite of cyanidin-3-*O*-β-glucoside [113] and inhibits VCAM-1 and ICAM-1 expression in vivo [177], meaning that anthocyanins help to suppress the endothelial cell activation promoted by the retention of apoB-containing lipoproteins (LDL, very low-density lipoprotein (VLDL), and apoE remnants) in the subendothelial space. Lipoprotein accumulation leads to an increase in proinflammatory receptors (Toll-like receptor 2) and cytokines (MCP-1 and interleukins, IL) [172]. Daily Chinese cabbage anthocyanin-rich extract administration decreases inflammatory cytokines that, together with the mentioned reduction in adhesion molecule expression, leads to the inhibition of plaque formation in the arteries of hyperlipidemic mice [176]. Anthocyanins of rich purple sweet potato reduce oxidative stress markers levels, e.g., thiobarbituric acid-reactive substances, in the liver and kidney and lipid peroxide in plasma. Anthocyanins’ antioxidant effect is important to reverse the increase in ROS production endorsed by endothelial cell activation. The 50% decrease in the atherosclerotic plaque area in the aorta and the smaller plaque area in the aortic sinus observed in mice correlates with the VCAM-1 decrease and the enhancement of antioxidant effects promoted by anthocyanins from purple sweet potato [175]. The association between anthocyanins’ antioxidant effects and aortic lesion suppression is also supported by a study in which mice were fed daily with a diet formulated to contain 1% freeze-dried whole blueberries. The upregulation of the genes of some antioxidative enzymes in the aorta was accompanied by the decrease in hepatic lipid peroxidation [178]. Similarly, a nutritional dose of bilberry anthocyanin-rich extract supplementation affects aortic genes expression encoding proteins involved in oxidative stress, inflammation, transendothelial migration and angiogenesis [179]. A different study showed that, in an early atherosclerosis stage, the expression of several hepatic genes encoding proteins involved in lipid metabolism and inflammation are also modified by anthocyanin-rich bilberries. According to the authors, this hepatic gene expression modulation could explain the reduction in plasma cholesterol level observed, probably due to an increased excretion as bile acid. Gene expression modulation may also indicate that the reduction in triglyceride (TG) level observed in the liver is due to the decrease in hepatic lipogenesis. Regarding pro-inflammatory genes, a down-regulation of their expression in the liver may also protect against atherosclerosis [180]. Anthocyanins reduce LDL oxidation both by increasing plasma protective enzyme activity such as that of paraoxonase 1 (PON1) [181,182], and decreasing the activity of enzymes such as the inducible nitric oxide synthase (iNOS), that generates strong oxidants that can readily oxidize LDL [183]. In arteries walls, monocytes differentiate into macrophages that can uptake oxLDL and form foam cells. Monocyte recruitment is facilitated by the presence of neutrophils, which are the most abundant leucocytes recruited to the inflammation site [172,173]. Treatment with both cyanidin-3-*O*-β-glucoside and PCA reduces monocyte/macrophage infiltration, at least in part, via negatively modulating the expression of chemokine receptor 2 [184]. Diet supplementation with cyanidin-3-*O*-β-glucoside induces ATP-binding cassette transporter G1 (ABCG1) expression and decreases cholesterol and 7-ketocholesterol (7-KC) accumulation in the aorta, which suggests that ABCG1 induction can improve vascular relaxation by reducing cholesterol and 7-KC accumulation. The hypocholesterolemic potential of cyanidin-3-*O*-β-glucoside can be partially achieved via the increment of fecal bile acid excretion arising from activating the potential liver X receptor alpha (LXRα)-CYP7A1-bile acid excretion pathway [185]. PCA induces ABCG1 and ATP-binding cassette transporter A1 expression in macrophages by decreasing the expression of miR-10b. This could contribute to accelerate macrophage reverse cholesterol transport, which can, at least in part, result in the regression of established atherosclerosis [186]. Anthocyanin-rich extract from black rice reduces serum total cholesterol (TC) and non-high-density lipoprotein (HDL) cholesterol, resulting in an enhancement of atherosclerotic plaque stabilization [183]. Anthocyanin-rich black elderberry extract supplementation results in changes in hepatic gene expression related to HDL function, and a reduction in aortic cholesterol [181]. A recent study shows that black elderberry extract also increases cholesterol efflux capacity and decreases liver inflammation markers. Interestingly, black elderberry extract feeding enhances deposition of connective tissue in aortic roots, which may positively promote plaque stability [182].

Other rodents are also used to study anthocyanins’ effects on atherosclerosis; these animal models are based on accelerated plaque formation due to a cholesterol-rich/Western-type diet. The supplementation of that diet with anthocyanins has an antiatherogenic effect, which is related to its antioxidant and antilipidemic actions, as observed in mice fed with roselle [187] and black highland barley [188]; in rats fed with extract from black rice [189], anthocyanin-rich red cabbage extract [190], ethanol extract of black mulberry [191], anthocyanin extract from *Nitraria tangutorun* Bobr. fruits [188] and chokeberry and purple maize [192]; hamsters fed with raspberry juices [193], roselle extract [194] and cranberry extract [195]; and also in guinea pigs fed with blueberries [196]. The cholesterol-lowering activity of anthocyanin extract could be mediated by the increase in the excretion of TC, TG and both total neutral and acidic sterols in feces, as observed in rats treated with anthocyanin-rich red cabbage extract and [190] and hamsters fed with cranberry extract [195]. The obese Zucker rat (OZR), a model of the metabolic syndrome with severe dyslipidemia, is also very useful to study atherosclerosis. When these rats were fed with a wild blueberry-enriched diet (8%), a decrease in TC concentration leading to a decrease in LDL cholesterol and/or in VLDL cholesterol was observed. However, no change was observed in HDL cholesterol concentration. This lipid profile induced by the wild blueberry ingestion suggests their role in the prevention of atherosclerosis [197]. Using a similar experimental design, a wild blueberry-enriched diet resulted in an MCP-1 concentration reduction in the perivascular adipose tissue of OZR, which also highlights their protective role regarding atherosclerosis [198].

New Zealand White rabbits fed a cholesterol-rich diet have been frequently used in atherosclerosis research, including in studies that examined the effects of anthocyanins. Using this model, it was observed that Concord grape juice decreases serum cholesterol, attenuates platelet aggregation and reduces aortic atheroma development [199]. In hypercholesterolemic rabbits, supplementation with *Anethum graveolens* powder decreases alanine aminotransferase, aspartate aminotransferase, TC, glucose, fibrinogen and LDL cholesterol [200]. Furthermore, a diet supplementation with hydroalcoholic extracts of *Amaranthus caudatus* reduces the risk factors and cause regression of fatty lesions in the aorta in rabbits [201]. Anthocyanins from the cornelian cherry have a positive impact on the serum lipids in cholesterol-fed rabbits. Anthocyanins increase the expression of peroxisome proliferator-activated receptors (PPARs), especially PPARγ [202]. Roselle extract, which contains anthocyanins, prevents LDL oxidation in the arterial wall of New Zealand White rabbits, showing potential to lower the incidence of atherosclerosis and coronary heart disease [203].

The zebrafish was also used as a model for studying the anti-atherosclerotic effects of three major sources of flavonoids and phenolic compounds including anthocyanins, namely loquat leaf, grape skin and acai puree. It was observed that all shared antioxidant, anti-inflammatory and anti-atherosclerotic activity in this hypercholesterolemic zebrafish model [204].

Regarding cardiac hypertrophy, fewer animal studies were conducted to evaluate the effects of anthocyanins. Treatment with roselle (composed by delphinidin-3-*O*-sambubioside and cyanidin-3-*O*-sambubioside) for 28 days ameliorates cardiac function, reduces cardiomyocyte hypertrophy and cardiac fibrosis, and attenuates cardiac oxidative stress in obesity alone as well as in obesity with myocardial infarction conditions. These protective effects of roselle were comparable to the enalapril angiotensin-converting-enzyme inhibitor [205]. The antihypertensive and cardioprotective effects of roselle extract were also shown in a study of 2-kidney-1-clip rats [206]. Reduction in cardiac cell inflammation, hypertrophy and attenuation of cardiac fibrosis are also obtained with the administration of purple-rice anthocyanin in streptozotocin-diabetic rats, suggesting a protective role against diabetic complications [207]. Both whole grape and red grape skin polyphenolic extract consumption reduced blood pressure, improved vascular function and compliance, and attenuated cardiac hypertrophy in the spontaneously hypertensive rat [208,209]. Treatment with cyanidin-3-*O*-β-glucoside for 15-week prevents cardiac hypertrophy and diastolic dysfunction but is unable to alleviate severe hypertension in spontaneously hypertensive rats, which means that cyanidin-3-*O*-β-glucoside may have direct cardioprotective effects independent of blood pressure [210]. This was confirmed by another study with obese C57BL/6 mice where, despite the fact that blueberries supplementation reduced both systolic and diastolic blood pressure, cyanidin-3-*O*-β-glucoside did not reduce blood pressure. Further investigation is needed to explore if other active components of blueberries or synergistic effects of multiple components are associated with the anti-hypertensive effect of blueberries [211]. Anthocyanins (cyanidin derivates) were the most abundant compounds found in a dried chokeberry fruit extract administered for 4 weeks to spontaneously hypertensive rats, and their antioxidant effects were associated with observed systolic and pulse pressure reductions [212]. In a high-fat diet mouse model, bilberries also can ameliorate or prevent metabolic disturbances associated with developing obesity, especially systemic low-grade inflammation and hypertension [213]. Chronic intake of alcohol-free red wine rich in anthocyanin malvidin 3-*O*-β-glucoside increases antioxidant capacity and reduces susceptibility of spontaneously hypertensive rats’ plasma to lipid peroxidation [214].

#### 5.1.2. Clinical Studies

Several randomized controlled trials have been carried out to find cause−effect relationships between anthocyanins and CVD prevention and treatment. Regarding atherosclerosis, and like in the animal studies, clinical trials also demonstrate that anthocyanins’ effects occur in different atherosclerotic stages. In areas of atherogenic lesions, the activation of endothelial cells can occur due to chronic infection, free radicals, hypertension, diabetes and cigarette smoking. This activation prompts the expression of genes such as MCP-1 mRNA, involved in induction of transcription factors responsible for shear stress-mediated effects. When overweight/obese individuals with impaired glucose intolerance or diabetes type 2 follow a diet supplemented with a standardized (36% (*w/w*) anthocyanins) concentrated bilberry extract, no changes in plasma levels of MCP-1 are observed. However, the ingestion of the bilberry extract reduces the postprandial glycemic response [215]. Anthocyanins isolated from berries were given to hypercholesterolemic individuals and an improvement in endothelium-dependent vasodilation through the activation of the nitric oxide–cyclic guanosine-3′,5′-monophosphate signaling pathway was observed [216].

A randomized, double-blind trial carried out on 150 subjects with hypercholesterolemia, consuming purified anthocyanin mixture (320 mg/day) or a placebo twice a day for 24 weeks, showed that anthocyanin consumption reduces serum levels of high sensitivity C-reactive protein (hsCRP) and plasma IL-1β compared to the placebo [217]. This could mean that anthocyanins reduce the inflammatory response activated by the interaction(s) between endothelial and white blood cells. The reduction in the inflammatory response reduces monocyte activation and, subsequently, decreases the affinity of monocyte ligands to adhesion molecules, as demonstrated by the decrease in plasma level of soluble VCAM-1. Purified anthocyanin supplementation for 24 weeks reduces serum levels of LDL cholesterol and increases HDL cholesterol levels in subjects with moderate hypercholesterolemia. Additionally, the change in LDL cholesterol positively correlates with the hsCRP change after 24-week anthocyanin intervention. The improvement in lipid profile sems to be correlated with the decrease observed in the serum levels of CRP, VCAM-1 and IL-1β in hypercholesterolemic subjects supplemented with an anthocyanin mixture to for 24 weeks [217]. Moreover, in hypercholesterolemic individuals, the decrease in LDL-C, hsCRP and IL-1β levels are correlated with the decrease in plasma levels of the platelet chemokines [218]. These pro-inflammatory molecules released by activated platelets mediate the pro-atherogenic effects that promote recruitment, activation or differentiation of other cell types including endothelial cells and leukocytes [219]. Anthocyanin supplementation (MEDOX^®^ capsules, 320 mg/day) for four-weeks also decreases blood levels of the proinflammatory cytokines’ tumor necrosis factor α (TNF-α), IL-6 and CCL2 in lean, overweight and obese populations [220]. Proinflammatory markers decrease with a bilberry-rich diet in individuals with features of metabolic syndrome. The bilberry-rich diet also promoted a differential regulation of the genes related to TLR signaling, cytoplasmic ribosomal proteins, and to the B-cell receptor signaling pathway as well as the differential expression of MMD (monocyte to macrophage differentiation associated) and CCR2 (CCL2 receptor) transcripts representing monocyte and macrophage function-associated genes [221].

Regarding anthocyanins’ effects on lipid profile, there are different results among clinical trials. Some report that consumption of anthocyanins does not produce favorable effects on lipoprotein concentrations of healthy subjects, as observed from the results that suggested that daily consumption of anthocyanins derived from blood orange juice for one month did not reduce LDL cholesterol nor any biomarkers associated with vascular function and CVD risk [222,223]. In addition, short-term (2 weeks) supplementation with cranberry juice in a group of young, healthy volunteers did not influence several biomarkers of blood lipid profile (TC, HDL and LDL) [224]. On other hand, there are many reports that suggest that anthocyanins decrease both LDL cholesterol [203,217,218,225,226,227,228,229,230] and TG [229,230,231] and increase HDL cholesterol [217,225,226,229,230,232,233]. Furthermore, when hypercholesterolemic subjects received 160 mg of anthocyanins twice daily or placebo (*n* = 61 of each group) for 24 weeks in a double-blind, randomized, placebo-controlled trial, anthocyanin supplementation also increased the activity of HDL-PON1 and cholesterol efflux capacity (20.0% increase) compared with the placebo group. Inhibition of HDL-PON1 activity strongly prevents HDL antioxidant effects and cholesterol efflux capacity attenuation [226]. Supplementation with purified anthocyanin at 0–320 mg/day over a 12-week period has beneficial effects on lipid profiles and cholesterol efflux capacity in a dose–response manner. Supplementation with 80 and 320 mg/day of anthocyanin can produce moderate and strong improvements, respectively [234]. In conclusion, the results suggest that anthocyanin has a beneficial effect on the lipoprotein profile, which includes a decrease in LDL cholesterol and TG, and an increase in HDL cholesterol concentrations.

Daily intake of boysenberry juice is beneficial for reducing systolic blood pressure in subjects with higher systolic blood pressure, thus decreasing cardiovascular risk [235]. Anthocyanins present in roselle maybe also responsible for the antihypertensive effect of this herb, as observed in a double-blind, lisinopril-controlled clinical trial involving 171 hypertensive patients for 4 weeks [236], as well as when administered to patients with metabolic syndrome (125 mg/kg/day for 4 w) [237].

In summary, animal and clinical studies suggest that anthocyanins could reduce oxidative stress and ameliorate inflammation, being a potential, safe candidate for prevention and therapy of CVDs.

### 5.2. Neurodegenerative Diseases

Neurodegenerative diseases, such as Alzheimer’s disease (AD), Huntington’s disease (HD), Parkinson’s disease (PD), prion disease, and amyotrophic lateral sclerosis (ALS), are a group of disorders that share the abnormal accumulation of intraneuronal or extraneuronal misfolded/unfolded proteins. With an ageing population, concerns about neurodegenerative diseases are becoming an increasingly relevant topic. Multiple pathways, including apoptosis, autophagy, mitochondrial dysfunction or oxidative DNA damage and repair, have been identified in different neurodegenerative diseases; however, the functional mechanistic context in each disease is different. In fact, the mechanisms of neurodegenerative diseases are still far from being clarified, which is a major challenge for the discovery of a potential therapy that can help to delay the effects of aging and prevent these diseases. Anthocyanins have now become a topic of interest as a natural preventive/therapeutic strategy because they have the ability to protect neurons against oxidative stress, suppress neuroinflammation and modulate cell signaling pathways.

#### 5.2.1. In Vivo

The brain is highly vulnerable to oxidative stress, since has a high oxygen demand, consuming more oxygen than other body parts. Moreover, the brain is also rich in redox-active metals (copper and iron) that contribute to ROS generation. Brain membranes are composed of polyunsaturated fatty acids that are more susceptible to lipid peroxidation. Regulation of cellular ROS metabolism depends on several proteins involved in the redox mechanism with the phosphatidyl-inositol-3-kinase (PI3K)/AKT signaling pathway. Intracellular ROS generation mediates the PI3K/AKT pathway, which is the key cause of cell senescence and apoptosis [238]. Korean black bean anthocyanins reduced neurodegeneration in an APP/PS1 transgenic mouse model of AD. Anthocyanins from Korean black bean reduced ROS and inhibited the apoptosis controlled by the PI3K/Akt/GSK3 pathway (GSK3, glycogen synthase kinase 3), which activates the endogenous antioxidant nuclear factor erythroid 2–related factor 2/heme oxygenase-1 (Nrf2/HO-1) pathway and its target genes [239]. Additionally, anthocyanin-loaded polyethylene glycol-gold nanoparticles (PEG-AuNPs) regulate the p-PI3K/p-Akt/p-GSK3β pathway in the amyloid beta (Aβ)1–42 mouse model of Alzheimer’s disease. This regulation prevented the appearance of the tau protein during the hyperphosphorylation of serines 413 and 404 [240]. Blueberry attenuates radiation-induced oxidative stress in rats through attenuation of nicotinamide adenine dinucleotide phosphate as well as activation of Nrf2 [241]. Anthocyanin administration to a lipopolysaccharide (LPS)-induced neurotoxicity animal model (24 mg/kg/day for 2 weeks: 1 week before and 1 week co-treated with LPS) increased the level of p-Akt and p-GSK3β survival proteins [242]. Overall, these results suggest that anthocyanins reduce the extent to which ROS facilitate the functioning of the PI3K/AKT pathway and, therefore, have a neuroprotection effect by preventing apoptosis. Moreover, ingestion of *Myrciaria jaboticaba* berry peel berry peel minimizes GSK3β-mediated tau phosphorylation in the hippocampus of 3-week-old weaned male Swiss mice, corroborating better learning/memory performance [243]. The antioxidant effect of anthocyanins in the brain is also confirmed in animal studies through oxidative biomarkers levels. Malondialdehyde (MDA) content decreases with anthocyanin treatment, as with black chokeberry extract in the d-galactose mouse model [244], with cyanidin-3-*O*-galactoside or blueberry extracts in senescence-accelerated mice prone 8 (SAMP8) mice [245], with pelargonidin in the amyloid β_25–35_ (Aβ) rat model of AD [246] and with anthocyanins extracted from Korean black soybeans [247] and from bilberry [248] in LPS-injected mice. In the latter animal model, restoration of the levels of heat shock protein 70 after blueberry supplementation was also observed [249]. Additionally, anthocyanins are effective in the activation of endogenous antioxidant enzymes, namely superoxide dismutase and glutathione peroxidase [241,244,245].

Anthocyanins’ neuroprotector effects are also correlated with neuroinflammation mediation, which usually results from the anormal accumulation of aggregated host proteins that can activate inflammasomes. The prominent innate immune cells in the brain for inflammasome activation are microglia; however, additional resident cell types as astrocytes and neurons can also express and activate inflammasomes [250]. Bilberry anthocyanin consumption (20 mg/kg/day) activates astrocytes and microglia and improves their beta-amyloid protein plaques’ phagocytotic function in APP/PSEN1 mice. Bilberry anthocyanin consumption upregulates mRNA level of TYROBP, a microglial molecule linked to both TREM2 (triggering receptor expressed on myeloid cells 2) and CD33 (immunoglobulin-like microglial-surface) [251], and downregulates mRNA level of CX3CR1 (C-X3-C motif chemokine receptor 1), thus altering the phagocytotic function of microglia. Therefore, these data suggest that anthocyanin consumption can be a preventive and therapeutic approach targeting the CD33/TREM2/TYROBP signaling pathway in microglia [252]. Activation of c-Jun-N-terminal kinase (JNK) in the brain is involved in microglia activation and induction of proinflammatory cytokine genes coding for TNF-α, IL-6, or MCP-1 in addition to cyclooxygenase-2 [253]. Anthocyanin administration to LPS-treated mice reverses JNK activation and decreases the levels of the following inflammatory markers: nuclear factor kappa B, TNF-α and IL-1β [242,247,248]. Moreover, anthocyanins reduced the overexpression of these inflammatory markers and lowered JNK levels in d-galactose (d-gal)-treated rats [244,254,255]. In this animal model, anthocyanins also suppressed microgliosis and astrocytosis [254,255]. Similarly, the pathway p-JNK/NF-κB/p-GSK3β is inhibited by anthocyanins alone or conjugated with PEG-AuNPs, reducing the neuroinflammatory markers in the Aβ1-42-induced mouse model [256]. Blueberry reduces cyclooxygenase-2 expression levels in both the hippocampus and frontal cortex of rats exposed to ^56^Fe, which also shows the potential of anthocyanins to attenuate neuroinflammation [241]. Other studies suggest that anthocyanins could act as inhibitors of the activity of acetylcholinesterase (AChE) by lowering Aβ toxicity in the brain, thus causing a reduction in AChE [246,257].

Animal models fed with a high-fat diet have also been used to study anthocyanins’ neuroprotection, since fat was found to be a significant risk factor for the development of neurodegenerative diseases [258]. Under high-fat conditions, blackberry extract intake prevents the negative effects of neuroinflammation, since a decrease in the cytokine-induced neutrophil chemoattractant, the ciliary neurotrophic factor, the platelet-derived growth factor, IL-10, the tissue inhibitor of metalloproteinase and the receptor for advanced glycation end-products was observed in the brain of Wistar rats [259]. The addition of blueberry to a high-fat diet is possible to reverse some behavioral deficits of mice promoted by that diet, specifically, deficits in object recognition memory [260]. These neuroprotective effects of blueberry may be related with an attenuation in microglia activation and an increase in neuroplasticity [261]. Purple sweet potato color treatment improves AMP-activated protein kinase-mediated autophagy, further blocking oxidative stress and restoring hippocampal brain-derived neurotrophic factor protein levels, and ultimately suppressing hippocampal apoptosis [262]. A different study showed that purple sweet potato color treatment also decreases the expression of cyclooxygenase-2, iNOS, TNF-α, IL-1β and IL-6, and increases the level of IL-10 in the brain of a high-fat diet-treated mouse. The authors suggested that purple sweet potato color treatment attenuated neuroinflammation induced by the high-fat diet by inhibiting extracellular signal-regulated kinase, JNK, p38 and NF-κB activation [263]. More recently, it was shown that supplementation of anthocyanin-rich *Syzygium malaccense* fruit in mice fed a high-fat diet improves antioxidant defenses and peripheral and hippocampal lower phosphorylation of tau [264].

Regardless of the mechanism behind the neuroprotector effects of anthocyanins, they result in memory improvement. This enhancement is confirmed by Western blot analysis of the memory-associated presynaptic and postsynaptic protein markers [239,240] and by behavioral tests. Anthocyanins improve learning and memory, which has been confirmed by the Morris water maze [240,243,244,245,252,253,254,260,264,265,266], Y-maze [240,255], novel object recognition [241,248,260,265] and passive avoidance [245,254,263] tests. Motor coordination is also improved by anthocyanins as confirmed by rotating rod test [244] and open-field [248,263] tests.

#### 5.2.2. Clinical Studies

Consumption of 200 mL/day of cherry juice by adults older than 70 years with mild-to-moderate dementia leads to an improvement in verbal fluency, short-term memory and long-term memory. Anthocyanin-rich juice promote a decrease in systolic blood pressure. Inflammatory markers (CRP and IL-6) were not altered by this intervention [267]. Concord grape juice [268] and wild blueberry juice [269] consumption has also potential to improve cognitive function in older adults with early memory decline. A randomized, double-blind, placebo-controlled trial of 24 weeks with elderly subjects who had mild self-perceived cognitive decline with aging showed that supplementation with fish oil and with blueberry reduces self-reported inefficiencies in everyday functioning [270]. Anthocyanins’ neurocognitive benefit was also confirmed by functional magnetic resonance imaging in a study where blueberry diet supplementation enhanced neural responses during working memory challenges in older adults with cognitive decline [271]. Improvement in attention/working memory performances was also observed in individuals undergoing mild cognitive decline by consuming table grapes twice a day [272]. Moreover, anthocyanins improve brain perfusion and activation in brain areas associated with cognitive function in healthy older adults supplemented with blueberries [273] and with *Vitis vinifera* fruit extract [274].

Studies with young healthy adults are controversial; some suggest that anthocyanins have no effect on cognition [275] and others found a cognitive benefit [276,277]. Regarding children, it was found that anthocyanin supplementation improves cognitive performance of 7–10-year-old children [278,279].

In summary, animal studies and random clinical trials suggest that anthocyanins improve cognition and neuroprotection. According to in vivo studies, the mechanisms responsible for these benefits are related with anthocyanins’ ability to decrease brain oxidative stress, inflammation and degeneration. Further research must focus on finding the dose and the frequency of the treatment with anthocyanins to be applied to humans to attain neuroprotector benefits.

## 6. Conclusions

Anthocyanins are colored molecules widespread in nature that display a wide array of beneficial effects on human health. An adequate daily intake of these substances may provide protection from numerous disease and disorders, particularly neurodegenerative and cardiovascular diseases. The chemistry and biochemistry of these compounds have been deeply investigated during the past years, with the aim to improve their stability and to use them as colorants or additives to supply nutritional values to foods. New nanoformulations or encapsulation of anthocyanins, along with chemical modifications such as acylation, provide more stable anthocyanins that may be useful in nutraceutical products. These formulations may also improve anthocyanins’ bioavailability, thus further improving their beneficial effects on health. Further studies devoted to the elucidation of the mechanism of disease prevention are needed. The protective effects of anthocyanins in CVDs and neurodegenerative diseases are similar and related mainly with their antioxidant and anti-inflammatory properties. In order to increase the knowledge about anthocyanins’ effects, it could be a good option to address together those age-related diseases, for example, using the apoE-deficient mouse—a widely used model of human atherosclerosis that has hyperlipidemia and develops all human atherosclerotic lesions. This model is also used to study AD.

## Abbreviations

ABA	Abscisic acid;
ABC	ATP-Binding cassette;
ABCG1	ATP-Binding cassette transporter G1;
ABTS+•	2,2′-Azino-bis(3-ethylbenzthiazoline-6-sulfonic acid radical;
AD	Alzheimer’s disease;
ALS	Amyotrophic lateral sclerosis;
ANS	Anthocyanidin synthase;
Apo	Apolipoprotein;
Aβ	Amyloid beta;
CCL2	C-C Motif chemokine ligand 2;
CCR2	CCL2 Receptor;
CD33	Immunoglobulin-like microglial-surface;
CHI	Chalcone isomerase;
CHS	Chalcone synthase;
CVDs	Cardiovascular diseases;
CX3CR1	C-X3-C Motif chemokine receptor 1;
DFR	Dihydroflavonol reductase;
DPPH•	D2,2-Diphenyl-1-picrylhydrazyl radical;
FRAP	Ferric reducing/antioxidant power;
F3H	Flavanone-3-hydrolase;
F3′H	Flavonoid-3′-hydroxylase;
FT-IR	Fourier transformed infrared spectroscopy;
GSK3	Glycogen synthase kinase 3;
HAT	Hydrogen atom transfer;
HD	Huntington’s disease;
HDL	High-density lipoprotein;
HO-1	Heme oxygenase-1;
HPLC	High performance liquid chromatography;
HR-MS/MS^n^	High resolution and tandem mass spectrometry;
HSCCC	High-speed counter-current chromatography;
iNOS	Inducible nitric oxide synthase;
ICAM-1	Intercellular adhesion molecule-1;
IL	Interleukin;
JNK	c-Jun-N-terminal kinase;
7-KC	7-Ketocholesterol;
LDL	Low-density lipoprotein;
LPS	Lipopolysaccharide;
MDA	Malondialdehyde;
MCP-1	Monocyte chemoattractant protein-1;
MMD	Monocyte to macrophage differentiation associated;
MPK	MAP Kinase;
NADES	Natural deep eutectic solvents;
NBT	Nitro blue tetrazolium;
NMR	Nuclear magnetic resonance;
Nrf2	Nuclear factor erythroid 2–related factor 2;
oxLDL	Oxidized LDL;
OZR	Obese Zucker rat;
PAL	Phenylalanine ammonia-lyase;
PCA	Protocatechuic acid;
PD	Parkinson’s disease;
PEG-AuNPs	Anthocyanin-loaded polyethylene glycol-gold nanoparticles;
PI3K	Phosphatidyl-inositol-3-kinase;
PON1	Paraoxonase 1;
PPARs	Peroxisome proliferator-activated receptors;
RNS	Reactive nitrogen species;
ROS	Reactive oxygen species;
SAMP8	Senescence-accelerated mice prone 8;
SCFA	Short chain fatty acids;
SET	Single electron transfer mechanism;
SPE	Solid phase extraction;
SPL	Squamosa-promoter binding protein-like;
TC	Total cholesterol;
TG	Triglyceride;
TLR2	Toll-like receptor 2,
TNF-α	Tumour necrosis factor α;
TREM2	Triggering receptor expressed on myeloid cells 2;
TT8	Transparent testa 8;
TTG1	Transparent testa glabra 1;
UFGT	UDP-Glucose:flavonoid-3-*O*-glycosyltransferase;
UPLC	Ultraperformance liquid chromatography;
VCAM-1	Vascular cell adhesion molecule-1;
VLDL	Very low-density lipoprotein.
